# The measurement of serum paracetamol using a discrete analyser

**DOI:** 10.1155/S1463924683000358

**Published:** 1983

**Authors:** R. Stewart Campbell, Peter M. Hammond, Michael D. Scawen, Christopher P. Price

**Affiliations:** 1 Department of Clinical Biochemistry Addenbrooke's Hospital Hills Road Cambridge CB2 2QR UK; 2 PHLS Centre for Applied Microbiology and Research Microbial Technology Laboratory Porton Down Salisbury SP4 0JG UK


Journal of Automatic Chemistry, Volume 5, Number 3 (July–September 1983), pages 146–149

### The measurement of serum paracetamol using a discrete analyser

**R. Stewart Campbell[1], Peter M. Hammond[2], Michael D. Scawen[2] and Christopher P. Price[1]**

*1. Department of Clinical Biochemistry, Addenbrooke's Hospital, Hills Road, Cambridge CB2 2QR, UK,*
*2. PHLS Centre for Applied Microbiology and Research, Microbial Technology Laboratory, Porton Down, Salisbury SP4 0JG, UK*

### Introduction

The current trend in clinical biochemistry towards the use of discrete analysers capable of performing a wide variety of analyses on a single sample, at the discretion of the analyst, suggests that this type of equipment may have an important role to play in the emergency laboratory. There are, however, few analysers possessing the facility for stat discretionary testing.

Recently, a specific method for the measurement of paracetamol has been described [1]. The method is a two-stage reaction involving, firstly, the enzymatic hydrolysis of the parent compound to p-aminophenol and acetate; and, secondly, the specific colorimetric detection of the p-aminophenol by the formation of a blue indophenol dye [2].

This assay has been adapted for use on a microcentrifugal analyser. In performing the adaptation the main objectives were to investigate (1) the limitations imposed by a two-stage reaction sequence; and (2) the performance of such an automated system.

### Materials and methods

### *Reagents*

All the reagents used were supplied in a diagnostic kit from Cambridge Life Sciences plc of Cambridge, UK. The enzyme reagent was a lyophilized preparation containing a buffer. Two colour reagents were supplied: colour reagent A was an aqueous solution of o-cresol, and colour reagent B was an ammoniacal copper sulphate solution. The kit also contained an aqueous paracetamol solution of 2·00 mmol/l as the standard.

### *Instrumentation*

The method was set up on a microcentrifugal analyser: the Multistat III (Instrumentation Laboratory, Lexington, USA). The instrument is capable of pre-mixing the sample and one reagent (to a combined maximum volume of 100 $\mu$l) in one compartment of a disposable rotor cuvette. A second reagent can be added to the second compartment (up to a maximum volume of 250 $\mu$l). The contents of the two compartments of the rotor cuvette are mixed when the rotor is spun in the analyser module. The cuvettes can be pre-warmed to 30°C or 37°C if required, and the absorbance reading can be made a minimum of 3 s after mixing.

### *Comparison method*

The results from the enzymatic method were compared with those from an HPLC method [3].

### *Automated method protocol*

The enzyme reagent was reconstituted by dissolving the contents of one bottle in 10 ml of deionized water. The bottle was stated to contain 10 units of enzyme activity. The colour reagents were mixed in a ratio of 1 : 1 to produce a combined colour reagent.

The loader was set up to dispense volumes of sample and enzyme reagent into one compartment of the rotor cuvette and combined colour reagent into the other compartment in volumes that, within the limitations imposed by the loader syringe capacities, matched as closely as possible the ratio of reagents used in the manual kit, i.e. 50 $\mu$l and 1 ml + 2 ml. The loader was set to mix 4 $\mu$l of sample with 79 $\mu$l of enzyme reagent and 1 $\mu$l of water (as a wash solution); 160 $\mu$l of combined colour reagent and 10 $\mu$l of water wash were dispensed into the second compartment.

### Experimental procedures and results

### *Reaction kinetics*

The original manual method was described as a two-stage reaction: an enzymatic degradation stage and a colorimetric detection stage. It was already known that the enzyme was irreversibly inhibited by copper ions [4]. However, the reaction kinetics were investigated for two protocols (1) the enzymatic stage proceeding to completion before mixing with the colour reagents; and (2) immediate mixing of all the reagents. The

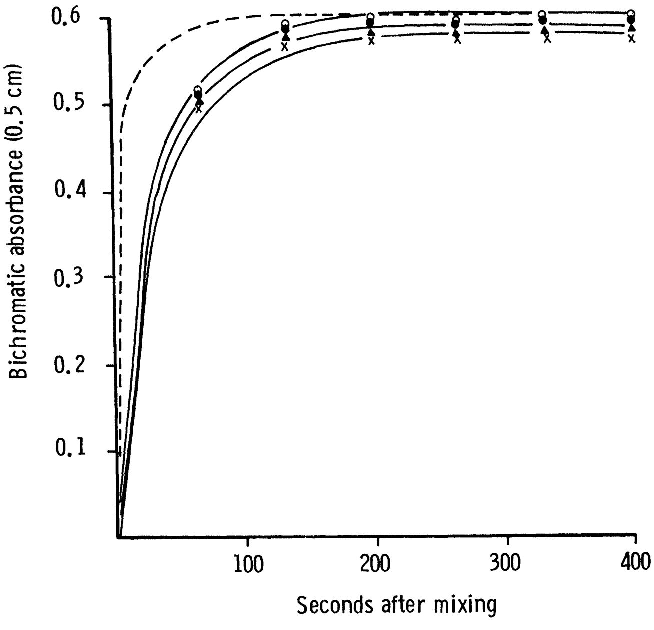

*Figure 1. Absorbance change due to the sample (a) pre-incubated with enzyme for 4 min before adding colour reagent (---) and (b) added to enzyme and colour reagent (——) according to the recommended protocol in cuvette 2 (○), cuvette 8 (●), cuvette 14 (▲) and cuvette 19 (×). The time taken to load one rotor of 20 samples was 8 min 40 s.*

absorbance changes were monitored for up to 5 min after mixing. The results show (see figure 1) that if the sample is pre-incubated with enzyme for 4 min, on addition of colour reagent the reaction proceeds to completion in under 100 s. If the sample is mixed with enzyme and immediately mixed with colour reagent the reaction is complete in less than 200 s. Clearly there is enough enzyme in the latter case to yield colour equal to that produced in the two-stage reaction.

### *Stability of enzyme/colour reagent mixtures*

The reagents were made up as specified by the manufacturer for performing the manual test. The reagents (enzyme reagent and two colour reagents) were mixed in the ratio 1 : 1 : 1 (v/v), and analysis of a serum and aqueous standard sample was performed immediately after mixing and at 10 min intervals for 1 h. The results (see figure 2) show that the enzyme activity is totally lost after the enzyme has been in contact with the colour reagents for 30 min.

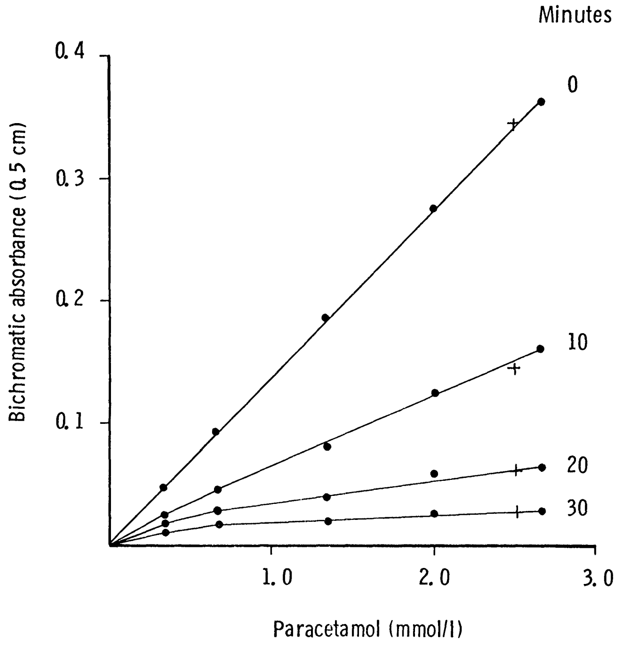

*Figure 2. The change in calibration curve using reagents in which the enzyme had been combined with the colour reagents for varying periods of time (0, 10, 20 and 30 min). The + denotes a serum sample containing paracetamol analysed at the same time.*

### *Stability of combined colour reagent*

The colour reagents supplied in the kit were mixed in the ratio 1 : 1 (v/v) and the assay protocol described was run regularly over a period of 72 h using a single batch of the combined colour reagent. The absorbance of the 2·0 mmol/l aqueous kit standard began to decrease 24 h after preparation of the combined colour reagent (see figure 3). So a combined colour reagent can be prepared for use over 24 h.

### *Stability of final colour*

The stability of the final assay colour produced by the protocol was established by preparing a standard curve with aqueous paracetamol standards. The standards were analysed in the assay, and readings of the final absorbance of the same rotor were taken at intervals up to 3 h after the initial reaction. The final colour was stable for at least 3 h at room temperature (see figure 4).

### *Linearity and choice of wavelengths*

A calibration curve was constructed using aqueous solutions of paracetamol up to a concentration of 3 mmol/l. The standards were analysed in the assay described and absorbance readings taken at the 500, 520, 550, 620 and 690 nm wavelengths. Maximum sensitivity and linearity was obtained using a bichromatic wavelength combination of 620/690 nm (see figure 5), 620 nm being the primary wavelength.

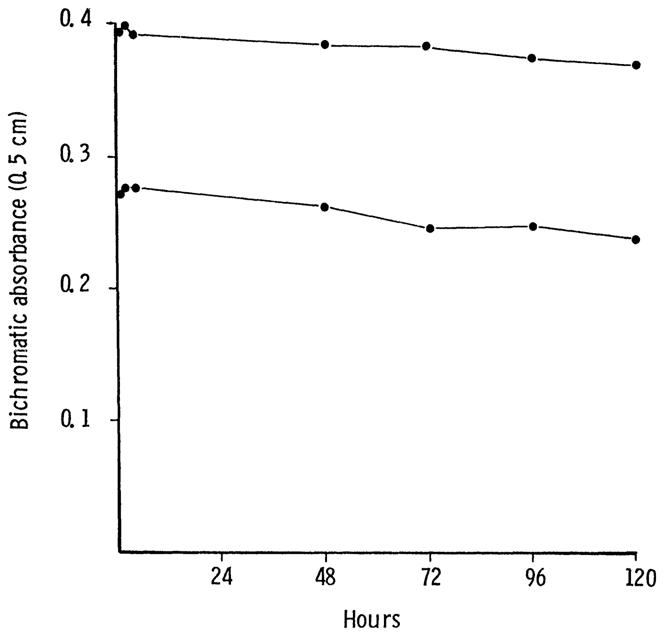

*Figure 3. The change in the absorbance produced when analysing two samples containing paracetamol where the colour reagents had been combined for varying periods of time.*

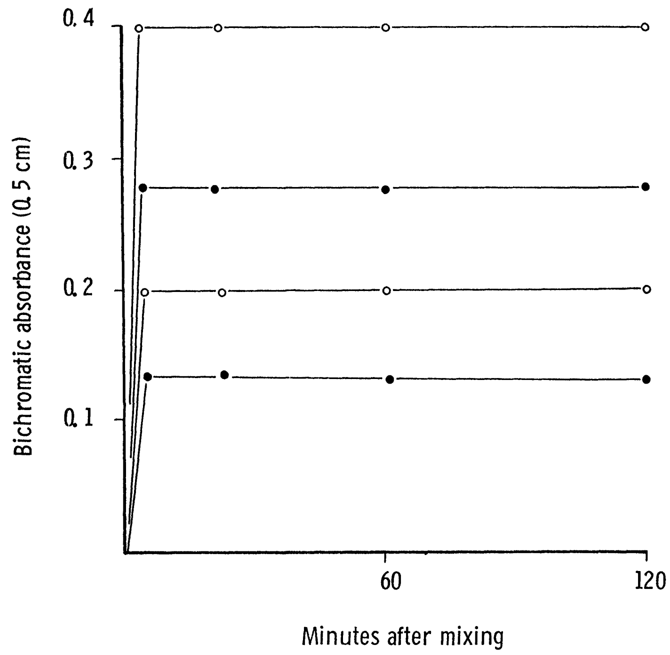

*Figure 4. The stability of the final colour produced when analysing two aqueous standards (○) amd two serum samples (●).*

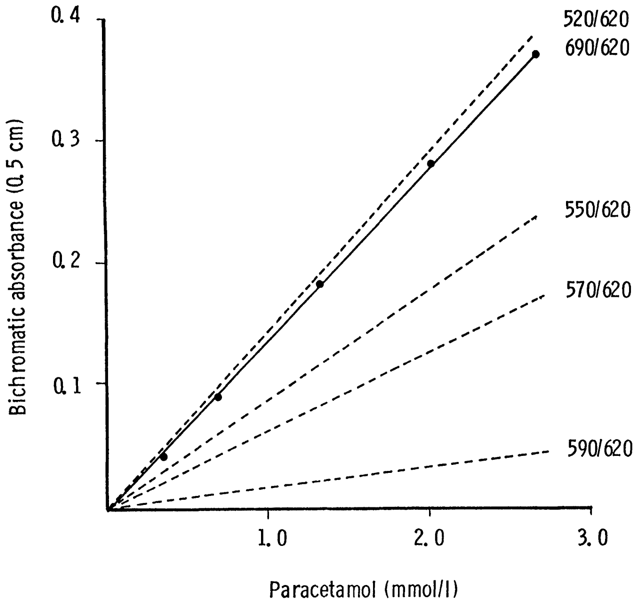

*Figure 5. The paracetamol calibration curve at various combinations of primary and blanking wavelength to indicate the linearity and relative sensitivity of the chosen system.*

### *Precision and carry-over*

The precision of the method was assessed by analysing aliquots of two serum pools known to contain paracetamol. The intra-assay precision was assessed using 18 aliquots of each sample and the data are shown in table 1.

The inter-assay precision was assessed using 20 aliquots each of two samples known to contain paracetamol. All aliquots were stored at −20°C until analysed. Fresh combined colour reagent was prepared for each analytical run. The inter-assay precision data are also shown in table 1.

Carry-over was assessed following the procedure of Broughton *et al.* [5], using a sample containing a high concentration of paracetamol and a sample known not to contain paracetamol. The carry-over was less than 0·2% at paracetamol concentrations of 2·0 mmol/l and 0 mmol/l.

### *Accuracy*

The accuracy of the automated procedure was assessed by comparing the analysis of 61 serum samples known to contain paracetamol by the automated procedure and an HPLC procedure [3]. The results of the comparison are shown in figure 6.

### *Final protocol*

As a result of the experiments described, a protocol was produced whereby the enzyme and sample in one rotor compartment and the combined colour reagent in the other rotor compartment were mixed immediately and the absorbance of each cuvette taken when the reaction was complete. Absorbance readings were taken using the bichromatic approach with a blanking wavelength of 690 nm and a primary reading wavelength of 620 nm. The first absorbance reading was taken 240 s after mixing, with the second (620 nm) reading taken 30 s later. The contents of the cuvette were not pre-equilibrated to a given temperature. A reagent blank was employed in the first cuvette of each rotor and results were calculated by comparison of the absorbance produced by the aqueous standard and that produced by the patient sample. Using this protocol, determination of paracetamol in a single sample (together with a standard and a quality-control specimen) will take 6 min of analyser time.

*Table 1. Precision data at two levels for the automated enzymatic paracetamol assay.*

| *Within-batch precision* | | |
|---|---|---|
| Mean (mmol/l) | SD | CV (%) |
| 0·33 | 0·005 | 1·44 |
| 1·93 | 0·025 | 1·31 |
| *Between-batch precision* | | |
| Mean (mmol/l) | SD | CV (%) |
| 0·59 | 0·009 | 1·67 |
| 2·13 | 0·033 | 1·53 |

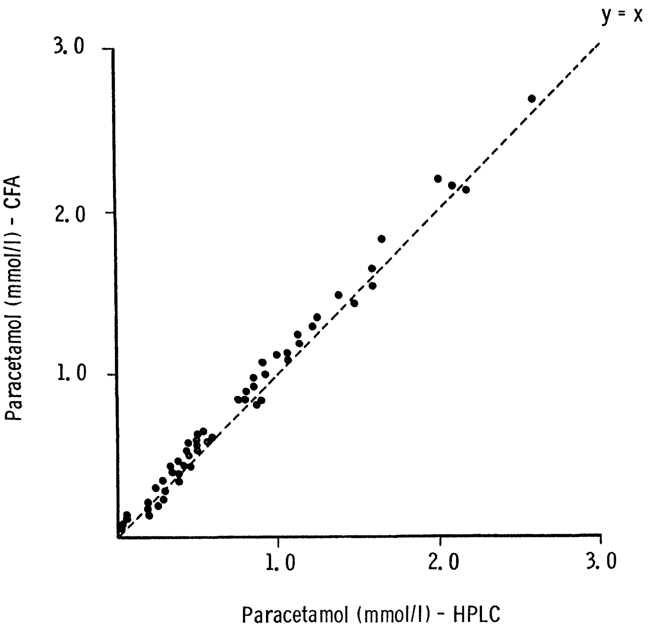

*Figure 6. Comparison of serum paracetamol results for patient samples analysed by the automated method and an HPLC method; the regression parameters are $y=1{\cdot}04x+0{\cdot}005$, $r=0{\cdot}996$, $N=61$.*

### Discussion

There are several methods available for the measurement of paracetamol [6]. The colorimetric methods lack the specificity required and have not been adapted to automated equipment. Spectrophotometric methods, although probably offering better specificity, are, because of the solvent extraction steps involved, unsuitable for automation. The chromatographic procedures offer the required specificity, however, sample preparation prior to chromatography may be time-consuming; they also demand technical expertise not always available on a 24 h basis. Hence, for some time there has been a need for a rapid, simple procedure for the specific determination of paracetamol.

The availability of an enzyme capable of degrading paracetamol has led to the development of a rapid two-stage assay, the specificity of the method lying in the substrate specificity of the enzyme and the highly specific colour reaction for the product of the enzymatic step, p-aminophenol. The assay is quick, specific, compares well with chromatographic reference methods [7] and has few manipulations; so it is well suited for use in an emergency laboratory.

The wide diversity of tests provided in an emergency laboratory requires the use of simple, rapid methods and simple instrumentation. In the past, the most appropriate instrumentation has been the single work-station approach with only one or two analytes being analysed on each instrument. However, with the increasing repertoire of tests needed by the clinicians, there is a call for an analyser that can handle several chemistries on a discretionary basis. To overcome the limitations this approach imposes on the methodology, and to gain maximum benefit from the use of a discretionary analyser, the number of analytical manipulations required of the analyser must be kept to a minimum. The enzymatic paracetamol assay has been successfully adapted to a discrete analyser that is capable of mixing a sample with up to two reagents. The reagents have been shown to be compatible in this environment and the precision and accuracy of the method are good. This suggests that the enzymatic paracetamol assay can be adapted to any discrete analyser that might be used in the emergency laboratory.

### References

1. Hammond, P. M., Scawen, M. D. and Price, C. P., *Lancet*, **1** (1981), 391.
2. Atkinson, A., Hammond, P. M., Price, C. P. and Scawen, M. D., UK Patent application 8136155 (1981).
3. Horvitz, R. A. and Jatlow, P. I., *Clinical Chemistry*, **23** (1977), 1596.
4. Hammond, P. M., Ph.D. thesis, University of Southampton (1982).
5. Broughton, P. M. G., Gowenlock, A. M., McCormack, J. J. and Neil, D. W., *Annals of Clinical Biochemistry*, **11** (1974), 207.
6. Weiner, K., *Annals of Clinical Biochemistry*, **15** (1978), 187.
7. Brown, S. S., Campbell, R. S., Price, C. P., Rambohul, E., Widdop, B., Barbour, H. M., Roberts, J. G., Burnett, D., Atkinson, T., Scawen, M. D. and Hammond, P. M., *Annals of Clinical Biochemistry* (in press).

### P.S. ANALYTICAL LTD LAUNCHED IN JULY 1983

Peter Stockwell has recently resigned from Plasma-Therm Ltd to set up his own marketing and manufacturing company: P.S. Analytical.

The new company has entered into an exclusive agency agreement with Plasma-Therm to market the Automated Hydride Generator System throughout Europe. The Hydride Unit has been successfully coupled to a wide range of Inductively Coupled Plasma Emission Spectrometers and, more recently. Atomic Absorption Spectrometers. It provides fast, reliable trouble-free analysis of hydride-forming elements and for mercury in the sub ppb level.

To complement this unit a large-volume autosampler is also available. The unit, which as standard will accommodate twenty 50 ml sample bottles, can be linked to the hydride unit and/or a remote computer. Different turntable configurations are available and the unit has a wide range of applications.

In addition to developing new automated analytical instruments. P.S. Analytical will also market a range of Teflon rotary valves and a fully automated HPLC injection unit.

P.S. Analytical will operate directly in the UK and through a series of established agencies in Europe.

*For further information, please contact P.S. Analytical Ltd, 2 Eagles Drive, Tatsfield, Westerham, Kent, UK. Tel.: Biggin Hill (09594) 73819. Telex: 8951183 Gecoms G.*

**Circle No. 1 on Reader Enquiry Card**

